# Annotations

**Published:** 1909-05-08

**Authors:** 


### ANNOTATIONS.

### Dichotomy.

Englishmen have got so much into the habit of regarding the United States of America as the home of all kinds of "graft" and corruption that no one would express great astonishment if it should appear that the prevailing epidemic has affected the morals of the medical profession over there. Whether this is so, or whether our American cousins are more outspoken and less hypocritical than ourselves is difficult to decide; but at least there is much more discussion in the medical press of the United States on these subjects than ever is printed in the United Kingdom. A botanical term has been borrowed to express one form of illicit business—"dichotomy," to wit, which is used as meaning the division of a surgeon's or specialist's fee with the practitioner who recommends him a patient. Now, a secret commission is an immoral thing, however its nature is disguised in lengthy euphemisms: the concealment of the transaction from the patient is the essential element of immorality about it. This practice, the editor of the *Medical Record* (New York) believes, is still the exception, not the rule, in America. But the mere fact that he feels constrained to protest against "dichotomy" being called a custom goes far to prove that it is not so very uncommon. No doubt the explanation is quite correct, that the practice is an attempt to remedy the gross inequality by which an operator who performs in half an hour or an hour some quite easy operation is paid without a murmur a fee such as the physician who watches over a case of typhoid for weeks is lucky indeed to receive. But however true the fact is that inequalities of remuneration for medical services exist, the way to remedy them is emphatically not by any form of secret commission or dichotomy.

### Medical Aspects of the Budget.

We gather from the reports in the newspapers of the Chancellor of the Exchequer's Budget speech that medical men are not to share in the reduction of the new petrol duty allowed to motor omnibus companies, taximeter cabs, and similar users of this spirit for industrial or commercial purposes. Yet the justice of preferential treatment for the medical profession as compared with the generality of motor car owners is tacitly admitted by the proviso that doctors' cars are to pay but half the taxes imposed on cars according to their horse power. Possibly by the time these lines appear in print some effort may have been made to ascertain precisely what the Budget actually does import in connection with the petrol duty, and to persuade the Chancellor to treat the practitioner at least as favourably as the proprietors of taxicabs and brewers' lorries. Another point in which the Budget will interest those members of the profession who dispense their own prescriptions is in regard to the heavy extra duty on spirits, which will inevitably raise the price of tinctures, and other pharmacological preparations involving this menstruum, and also that of products such as chloroform and ether. As for the latter, the accentuation of the already enormous difference in price between the drugs made from ethyl-alcohol and those from methylated spirit will probably still further diminish the relative popularity of the former, especially as recent improvements in manufacture have made it as impossible to distinguish them from one another chemically, as it already is clinically. As for alcoholic tinctures, the tendency to order watery tinctures, or watery solutions of the active alkaloid whose effect is required, will certainly be accelerated. This of itself will in many cases be no cause for regret, but there are others in which the most thoughtful physicians still believe in the superior virtues of the old-established alcoholic tinctures. Since one avowed object of these Budget proposals is to diminish alcoholic consumption as a luxury and as a vice, it seems not unreasonable that it should be pointed out to Mr. Lloyd George that medicines are neither luxuries nor vehicles for illicit dram-drinking; and that they are as much a necessity for the poor as for the rich. We would appeal, if it is not too late, to some of the medical men in Parliament to put the foregoing considerations before the minister responsible for the Budget of 1909.

### Metropolitan Ambulances Bill.

Sir William Collins on the 27th ultimo introduced into the House of Commons a short bill conferring powers on the London County Council to enable it to provide for London such ambulance service as the Departmental Committee's investigations have shown to be required. In an article published in The Hospital on the 3rd ultimo, page 21, we drew attention to the fact that the Departmental Committee appointed to inquire as to the provision made for dealing with cases of accident and sudden illness occurring in streets and public places within the metropolis was divided in opinion as to the authority in which the management and maintenance of the ambulance system should vest. A majority were in favour of giving power to the Metropolitan Asylums Board, but, as we pointed out at the time, Sir William Collins' protest against this recommendation was wisely made and ought to be generally supported by everybody interested in making London an up-to-date city replete with every modern contrivance for the benefit of its inhabitants. It virtually reproduces the powers sought by the London County Council three years ago, which, after examination by the Police and Sanitary Committee, were passed by the House of Commons but did not pass the Lords. It is far better to have a short act establishing an ambulance service in London and conferring powers on the London County Council, the proper authority, to maintain it efficiently. We therefore are strongly in favour of the bill, and hope that the House of Commons and the House of Lords will permit every facility to be given to expedite its passage during the present session of Parliament. Sir William Collins ought to have the support of all parties in securing this result.

